# Impact of Intraprocedural Pulse Pressure During Mechanical Thrombectomy on Functional and Radiological Outcomes in Patients with Acute Ischemic Stroke

**DOI:** 10.3390/medsci14010082

**Published:** 2026-02-11

**Authors:** Marcin Wiącek, Izabella Tomaszewska-Lampart, Halina Bartosik-Psujek

**Affiliations:** Department of Neurology, Faculty of Medicine, University of Rzeszow, 35-620 Rzeszow, Poland; itomaszewska@ur.edu.pl (I.T.-L.); hbartosik@ur.edu.pl (H.B.-P.)

**Keywords:** acute ischemic stroke, mechanical thrombectomy, endovascular treatment, pulse pressure, blood pressure, general anesthesia

## Abstract

**Background/Objectives**: Periprocedural blood pressure influences outcomes after endovascular treatment (EVT), but the impact of pulse pressure (PP) remains unclear. We assessed associations between intraprocedural PP and clinical and radiological outcomes after EVT. **Methods**: We retrospectively analyzed adults with acute ischemic stroke (AIS) due to proximal anterior circulation large vessel occlusion treated with EVT under general anesthesia. Non-invasive BP was recorded every 5 min during EVT. From these recordings, we derived baseline, maximal, minimal, and median PP, PP variability indices, and cumulative time above predefined PP thresholds. The primary endpoint was poor functional outcome at 90 days (modified Rankin Scale 3–6). Secondary endpoints were final infarct volume (FIV), malignant brain edema (MBE), symptomatic intracranial hemorrhage (sICH), and hemorrhagic transformation (HT). Multivariable regression models were adjusted for established prognostic factors. **Results**: In the 217 patients included, higher median PP during EVT independently predicted poor functional outcome, larger FIV, MBE, and HT, but not sICH. Longer duration of PP > 50 mmHg was independently associated with poor outcome, MBE, and HT. Among other hemodynamic variables, only a >40% mean arterial pressure (MAP) drop from baseline independently predicted poor outcome. Adding median PP to the baseline multivariable model modestly increased its discriminative ability and significantly improved model fit. **Conclusions**: In AIS patients with proximal anterior circulation large vessel occlusion treated with EVT under general anesthesia, higher intraprocedural PP and longer exposure to elevated PP are associated with worse functional and radiological outcomes, supporting PP as a potential hemodynamic target alongside avoidance of large MAP reductions.

## 1. Introduction

Despite successful reperfusion rates approaching 90%, only about half of the patients undergoing endovascular treatment (EVT) for acute ischemic stroke (AIS) achieve functional independence [1]. Among the factors influencing outcomes, periprocedural hemodynamic parameters may be of particular importance because they are potentially modifiable. Multiple studies have shown that various intraprocedural blood pressure (BP) metrics during EVT are associated with outcomes in patients with large vessel occlusion (LVO) [2,3]. Intraprocedural hypotension—defined using different thresholds and metrics—has consistently been identified as an independent risk factor for unfavorable EVT outcomes, including lack of functional independence and 90-day mortality, larger final infarct volume (FIV), and major complications of mechanical thrombectomy (MT), such as malignant brain edema (MBE) and symptomatic intracranial hemorrhage (sICH) [4,5,6]. Likewise, intraprocedural hypertension and pulse pressure (PP) variability—but not PP level itself—have been linked to lower odds of 3-month functional independence [7,8].

High PP, considered a surrogate marker of arterial stiffness, has been consistently associated with adverse cardiovascular prognosis [9,10,11]. Less is known about the role of elevated PP in the acute phase of ischemic stroke. Several studies assessing BP in the days following stroke onset have reported associations with poor prognosis and mortality in both short-term and 3-month follow-up [12,13]. More recently, elevated PP at 24 h after EVT was reported as an independent risk factor for poor functional outcome and sICH, suggesting that mean PP may be a better predictor of outcomes after MT than systolic BP (SBP) [14]. Together with mechanistic considerations and reports in AIS patients treated with intravenous thrombolysis (IVT), these findings indicate that PP may be an important and under-recognized predictor of outcomes after reperfusion therapy [8,15].

Therefore, we conducted this study to evaluate the association between intraprocedural PP metrics and multiple MT-related outcomes. This analysis was motivated by the paucity of comparable data and by observations that patients undergoing EVT—particularly under general anesthesia (GA)—may be especially vulnerable to BP fluctuations and their potentially deleterious consequences.

## 2. Materials and Methods

### 2.1. Study Design and Patient Selection

**Figure 1:**
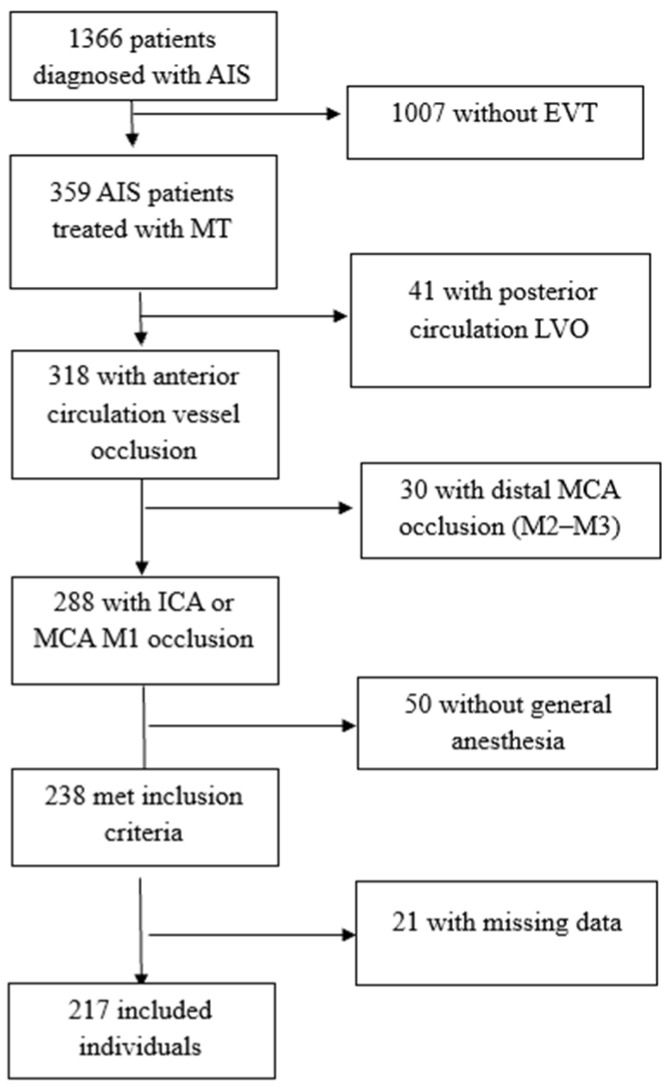
Flowchart of the inclusion of study population. AIS, acute ischemic stroke; EVT, endovascular treatment; MT, mechanical thrombectomy; LVO, large vessel occlusion; ICA, internal carotid artery; MCA, middle cerebral artery.

We conducted a retrospective analysis of consecutive patients diagnosed with AIS who underwent EVT for anterior circulation LVO at an academic comprehensive stroke center (Department of Neurology, Clinical Regional Hospital No. 2, Rzeszów, Poland). We enrolled a consecutive cohort of patients who underwent EVT over a 28-month period (between 1 December 2018 and 31 March 2021) and met the following inclusion criteria: (a) age ≥ 18 years; (b) occlusion of the intracranial internal carotid artery (ICA) and/or the proximal (M1) segment of the middle cerebral artery (MCA); and (c) EVT performed under GA. A flow diagram of patient selection is shown in Figure 1.

All MT-related decisions were taken by the endovascular team (an experienced neuroradiologist, a vascular neurologist, and an anesthesiologist) in accordance with the local protocol, the applicable European Stroke Organization (ESO) guidelines in force during the study period, and best clinical judgment [16,17]. Hypotension (SBP < 120 mmHg) was managed with ephedrine, phenylephrine, or norepinephrine at the discretion of the anesthesia team and hypertension (>180 mmHg) with urapidil at the anesthesia team’s discretion.

All procedures were performed at a comprehensive stroke center by four certified neurointerventionalists (three neuroradiologists and one neurologist), each with more than five years of EVT experience. The institutional annual EVT volume during the study period was approximately 170 cases.

The study was conducted in accordance with the Declaration of Helsinki and approved by the Ethics Committee of University of Rzeszow (No. 2022/50, date of approval 4 May 2022). Due to the observational nature of the analysis and the absence of any additional patient interventions, the requirement for written informed consent was waived.

### 2.2. Data Collection and Outcome Measures

Patient demographics, medical history, baseline clinical characteristics, and imaging findings were extracted from electronic medical records. Stroke etiology was categorized using the TOAST (Trial of Org 10172 in Acute Stroke Treatment) classification. LVO location was defined as the most proximal intracranial site of occlusion, and tandem occlusion was defined as concomitant extracranial ICA occlusion and intracranial arterial occlusion [17].

Blood pressure data were extracted from the intraprocedural anesthesia record. These data included systolic blood pressure (SBP), diastolic blood pressure (DBP), and mean arterial pressure (MAP) measured non-invasively before the EVT procedure and every 5 min until successful recanalization (or termination of the procedure in case of unsuccessful recanalization). Pulse pressure (PP) was calculated as the difference between SBP and DBP (PP = SBP − DBP) for each measurement. The following PP-derived variables were analyzed: baseline PP (the pre-procedural value), maximal PP (highest recorded PP during the procedure), minimal PP (lowest recorded PP), and median PP (median of all intraprocedural PP measurements). PP variability was assessed using the coefficient of variation (CV), calculated as the standard deviation divided by the mean PP for each patient. Short-term fluctuations were captured by the maximal successive change (MSC), defined as the largest absolute difference in PP between two consecutive measurements, and by the maximum–minimum difference (DMM), defined as the difference between the maximal and minimal PP values during the observation period. Additionally, we evaluated other BP parameters: (a) baseline SBP/DBP/MAP (defined as the value of the measurement directly preceding induction of anesthesia); (b) intraprocedural minimal SBP/DBP/MAP value; (c) intraprocedural maximal SBP/DBP/MAP drop below its baseline value; and (d) SBP/MAP max. drop >0%/>20%/>40% (yes/no).

In addition, we quantified the cumulative time with PP above predefined thresholds (>50, >60, >70 and >80 mmHg). For each threshold, the total time (minutes) was calculated by summing the measurement intervals during which PP exceeded the respective cutoff (each measurement interval was assumed to represent the subsequent 5 min period as described elsewhere [2]). These PP thresholds were not derived from the prior literature and were selected purely as exploratory, data-driven cutoffs intended to characterize the range of pulsatile fluctuations observed during EVT.

All patients underwent non-contrast CT (NCCT) imaging as part of routine follow-up 24–36 h after EVT. Symptomatic intracranial hemorrhage (sICH) was defined according to the Safe Implementation of Thrombolysis in Stroke–Monitoring Study (SITS-MOST) criteria [18]. Specifically, sICH was classified as parenchymal hematoma type 2 (PH2): hemorrhage involving >30% of the infarcted area with a substantial space-occupying effect, accompanied by a ≥4-point increase in the National Institutes of Health Stroke Scale (NIHSS) score. Cerebral edema (CED) was categorized as CED-0 (no edema), CED-1 (focal swelling involving less than one-third of the hemisphere), CED-2 (swelling involving more than one-third of the hemisphere), and CED-3 (edema with midline shift). In line with prior studies, malignant brain edema (MBE) was defined as CED-3 [6,19]. FIV was evaluated by two experienced vascular neurologists and calculated using a validated semi-automatic method (3D Slicer 4.10; http://www.slicer.org; accessed on 1 September 2025) as described elsewhere [5,20]. Post-MT reperfusion was graded using the modified Thrombolysis in Cerebral Infarction (mTICI) scale: grades 2b (perfusion > 50% of the vascular territory of the occluded artery), 2c (near-complete perfusion except for slow flow in a few distal cortical vessels or the presence of small distal cortical emboli), and 3 (complete perfusion with normal filling of all distal branches) were considered successful reperfusion [21]. Functional outcomes at 3 months were assessed using the modified Rankin Scale (mRS), either in person or by telephone, in accordance with our standard follow-up procedure; good outcome was defined as mRS 0–2 and poor outcome as mRS 3–6. Functional outcome was the primary endpoint, whereas FIV, MBE, sICH, and any hemorrhagic transformation (HT) were secondary endpoints in this study.

### 2.3. Statistical Analyses

Continuous variables are presented as mean ± standard deviation (SD) or median (interquartile range; IQR) depending on the normality of the distribution. Categorical variables were presented as numbers (percentages). Univariate analyses were performed using Fisher’s exact test for dichotomous variables and Student’s *t* test (two-tailed) or Mann–Whitney U-test for continuous variables, as appropriate. Associations between the analyzed parameters and categorical clinical and radiological outcomes were assessed using multivariable logistic regression models adjusted for predefined covariates selected based on the prior literature, univariate analyses, and clinical considerations, including admission NIHSS score, age > 80 years, hypertension, chronic heart failure, bridging thrombolysis, onset-to-groin time, groin-to-recanalization time, and successful recanalization. The categorical outcomes included poor functional outcome (mRS 3–6), MBE, any HT, and sICH. Associations between hemodynamic variables and FIV were evaluated using Spearman’s rank correlation and multivariable linear regression models adjusted for the same covariates. In addition to outcome-based group comparisons, all pulse pressure-derived metrics were analyzed as continuous exposure variables in both univariable and multivariable models. Group comparisons presented in Table 1 serve only as a descriptive characterization of the cohort and not as the primary analytic framework. To account for multiple testing in the univariable analyses, *p*-values from group comparisons and unadjusted PP–outcome associations were corrected using the Benjamini–Hochberg false-discovery rate method, and q-values are reported where applicable. No multiplicity correction was applied to multivariable regression models, as these represent single joint inferential frameworks with non-independent regression coefficients.

To assess discriminative performance, receiver operating characteristic (ROC) curves were constructed and the area under the curve (AUC) with 95% confidence intervals (CI) was calculated using DeLong’s method. Discrimination of the baseline multivariable model and the model additionally including median PP was compared by the paired DeLong test (difference in AUC). Model fit for nested models (with vs. without median PP) was additionally compared using the likelihood ratio test.

All statistics were computed using PQStat Software 1.8 (Poznan, Poland). Statistical significance was set at *p* < 0.05 (two-tailed).

## 3. Results

A total of 217 patients were included in the study. The median age was 72 years (IQR 65–79), 102 patients (47.0%) were female, and the mean NIHSS score at admission was 17.1 ± 5.1. The etiology of AIS was cardioembolic in 122 patients (56.2%), large-artery atherosclerosis in 24 (11.0%), and internal carotid artery (ICA) dissection in 5 (2.3%). One patient (0.5%) had another determined etiology (thrombophilia), while in 65 patients (30.0%), the etiology was classified as undetermined according to the TOAST classification, mainly due to the coexistence of two or more potential causes.

During EVT, 205 patients (94.5%) experienced an intraprocedural decrease in MAP relative to the pretreatment value. The median of maximal MAP reduction was 38 mmHg (IQR 25–54), corresponding to a median relative decrease of 36% (IQR 26–45) from baseline. The median FIV on post-treatment NCCT was 148 mL (IQR 24–203). Baseline characteristics of the study cohort and their associations with functional outcomes are summarized in Table 1.

In univariable analyses of PP parameters, several parameters were significantly associated with unfavorable clinical outcome, including maximal PP, median PP, and DMM (Table 1). In patients with sICH, significantly higher baseline PP (median 80 vs. 63 mmHg, *p* = 0.005), maximal PP (84 vs. 73 mmHg, *p* = 0.042), MSC (30 vs. 25 mmHg, *p* = 0.025), and DMM (45 vs. 35 mmHg, *p* = 0.040) were observed. Any HT was associated with higher maximal PP (80 vs. 70 mmHg, *p* = 0.005), higher median PP (50 vs. 45 mmHg, *p* = 0.008), and higher DMM (40 vs. 35 mmHg, *p* = 0.027). Only median PP was significantly associated with MBE (50 vs. 50 mmHg, *p* = 0.046). Additionally, larger FIV was observed in patients with higher baseline PP (*p* = 0.005), maximal PP (*p* = 0.006), MSC (*p* = 0.049), and DMM (*p* = 0.038). Prolonged exposure to PP > 50 mmHg was also associated with MBE (median 43 vs. 30 min, *p* = 0.027), and a longer duration above this threshold was observed in patients with any hemorrhagic transformation. However, several of these univariable associations did not retain statistical significance after adjustment for multiple comparisons using the Benjamini–Hochberg procedure and should therefore be interpreted as exploratory.

To complement the descriptive comparison by functional outcomes, an additional table (Table 2) summarizing the distribution of all PP-derived metrics across the entire cohort (rather than by mRS categories) was included to present PP-related data directly. Table 2 summarizes intraprocedural PP-derived metrics across outcome groups. Several PP metrics differed between patients with and without hemorrhagic transformation, whereas associations with MBE and sICH were less consistent after multiplicity adjustment.

In multivariable analyses, median PP during MT was an independent predictor of unfavorable 3-month functional outcome, MBE, larger FIV, and HT, but not sICH. Longer duration of PP > 50 mmHg was independently associated with mRS 3–6, MBE, and any intracranial hemorrhage after EVT. Increasing PP thresholds were linked to a higher risk of unfavorable functional outcome and HT. The results of multivariable analyses are presented in Table 3 and Table 4.

**Figure 2:**
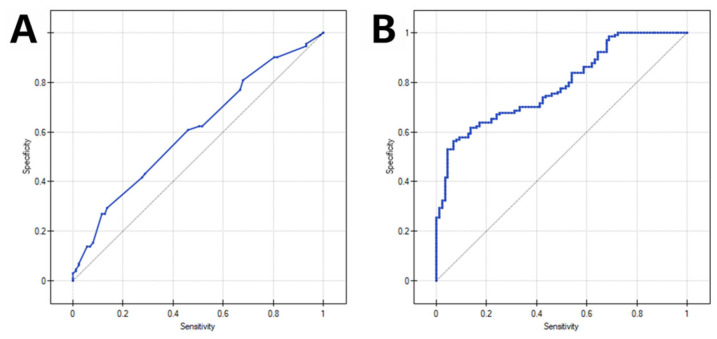
Receiver operating characteristic (ROC) curves for prediction of poor 90-day functional outcome. (**A**) Univariable model including median intraprocedural pulse pressure (PP) as the sole predictor. (**B**) Multivariable model adjusted for admission NIHSS score, age > 80 years, hypertension, chronic heart failure, bridging thrombolysis, onset-to-groin time, groin-to-recanalization time, and successful recanalization, with additional inclusion of median PP.

In a univariable model including median PP as the sole predictor of poor 3-month outcome, ROC analysis showed limited but statistically significant discrimination (AUC 0.613, 95% CI 0.536–0.690; *p* = 0.005). In the multivariable model adjusted for admission NIHSS score, age > 80 years, hypertension, chronic heart failure, bridging thrombolysis, onset-to-groin time, groin-to-recanalization time, and successful recanalization, discrimination was good (AUC 0.774, 95% CI 0.713–0.834; *p* < 0.001). After adding median PP to this baseline multivariable model, the AUC increased modestly to 0.789 (95% CI 0.730–0.847; *p* < 0.001); however, the improvement in discrimination was not statistically significant (ΔAUC = 0.015, *p* = 0.246). Conversely, inclusion of median PP significantly improved overall model fit (likelihood ratio test: χ^2^ = 6.74, df = 1, *p* = 0.009). ROC curves for the univariable and multivariable models incorporating median PP are shown in Figure 2.

Among hemodynamic variables other than PP, the following were associated with poor functional outcome: intraprocedural minimum DBP (median 55 vs. 55 mmHg, *p* = 0.022), a greater relative DBP decrease (43% vs. 40%, *p* = 0.018), a larger relative SBP decrease (38% vs. 35%, *p* = 0.046), and a MAP reduction > 40% from baseline (49 vs. 31% in the poor and good outcome groups, respectively; *p* = 0.012). After adjustment for potential confounders, only a MAP decrease >40% remained an independent predictor of poor outcome (aOR 2.01, 95% CI 1.07–4.1; *p* = 0.031).

## 4. Discussion

Our study demonstrated that median PP during MT was an independent predictor of poor 90-day functional outcome. Likewise, a longer cumulative duration of PP above 50, 60, and 80 mmHg was independently associated with lack of functional independence after EVT, with the 70 mmHg threshold narrowly missing statistical significance (aOR per 5 min above the threshold 1.03 95% CI 1.00–1.06, *p* = 0.086). It is noteworthy that the progressively higher PP thresholds were associated with increasing adjusted odds ratios for unfavorable outcome, further supporting a potential deleterious effect of elevated PP. These findings are consistent with previous reports identifying higher post-treatment PP as a predictor of poor functional outcome after EVT, with an optimal cut-off of 57.39 mmHg [14]. In contrast, a study focusing on the intraprocedural period found that PP variability, but not PP level alone, was associated with functional outcomes [8].

Our findings can be explained by the “biomechanical theory” of PP impact on brain microvasculature [9,22]. Pulsatile blood flow, shaped by stroke volume and wave reflections, is buffered by large elastic arteries, which absorb part of this “pulsatility” and help maintain relatively steady capillary flow in end organs such as the brain. With age-related increases in arterial stiffness, a greater pulsatile load may be transmitted to the cerebral circulation, potentially promoting microvascular stress, endothelial dysfunction, and downstream neuronal injury [22]. Excess pulsatile pressure may also contribute to blood–brain barrier (BBB) disruption with increased permeability [23]. Over time, these processes, driven by chronically elevated PP, may contribute to cerebral small vessel disease, including white matter hyperintensities, cerebral microbleeds, silent lacunar infarcts, chronic cerebral hypoperfusion, and neurodegeneration [24,25]. One plausible explanation linking PP to stroke outcomes is that such chronic vascular brain injury reduces “brain reserve,” thereby limiting the potential benefit of reperfusion therapy. It may also provide a background for microvascular injury in AIS due to LVO, as pre-existing endothelial damage and increased BBB permeability may increase the risk of treatment complications, such as HT and brain edema.

On the other hand, PP is not only dependent on arterial stiffness, but also on stroke volume, cardiac contractility, heart rate, and the timing and magnitude of wave reflections, suggesting that PP is influenced by more dynamic factors and could represent a potential target for modification. This may be particularly important in AIS due to LVO, where cerebral autoregulation can be impaired, making perfusion in vulnerable, collateral-dependent regions more dependent on systemic BP, so fluctuations in pulsatile pressure may have a greater impact on microcirculatory perfusion and secondary injury [26].

We observed that median PP during MT was positively correlated with FIV, and this association persisted after adjustment for potential confounders (aβ = 18.78, 95% CI 1.55–36.02, *p* = 0.033). Although no prior studies have directly reported this relationship, indirect support comes from reports linking higher post-stroke PP to early infarct progression and early neurological deterioration, processes that are closely related to infarct burden and final infarct volume [27,28]. These findings are also consistent with the proposed pathophysiological rationale. In AIS due to LVO, impaired cerebrovascular autoregulation may increase the dependence of cerebral blood flow (CBF) on systemic BP. In this setting, elevated PP could compromise collateral perfusion needed to sustain the penumbra until reperfusion, thereby promoting infarct expansion and larger FIV, which in turn may translate into worse clinical outcomes.

Additional results support a potential deleterious effect of elevated intraprocedural PP, given its associations with procedure-related complications. We found that higher intraprocedural PP was an independent predictor of MBE and HT, but not sICH. An association between PP and hemorrhagic complications of reperfusion therapy has also been reported in a study evaluating 24 h post-MT blood pressure, in which PP was linked to HT and sICH, with an adjusted odds ratio for sICH of 2.39 (95% CI 1.58–3.62), and in a cohort of IVT-treated patients, in whom admission PP was associated with HT in univariable analysis (*p* = 0.029) [14,28]. However, in the IVT cohort, this association did not remain significant after adjustment for confounders. The observed association between higher median PP during the acute phase of ischemic stroke and HT is consistent with the mechanisms outlined above, namely chronic microvascular and BBB vulnerability related to vascular aging and arterial stiffness, together with increased pulsatile stress on the collateral-dependent microcirculation during LVO and after reperfusion, which may facilitate HT. Notably, in our cohort the risk of HT after EVT increased with longer cumulative time spent above PP thresholds of 50, 60, 70, and 80 mmHg, with progressively higher adjusted odds ratios across thresholds. This supports the concept that single BP values are only surrogate measures of complex, time-dependent hemodynamic patterns during EVT, as emphasized in prior studies [2,5,8]. The lack of an association between PP and sICH in our study may reflect limited statistical power and, additionally, the strict SITS-MOST definition of sICH.

Another potential link between elevated PP and poor functional outcome, beyond its association with larger FIV and a higher risk of HT, is the development of MBE. In our cohort, higher intraprocedural PP was an independent predictor of this serious complication, with an approximately 40% increase in risk per 10-mmHg increase in mean PP (aOR per 10 mmHg increase in PP 1.39, CI 95% 1.03–1.86, *p* = 0.030). To the best of our knowledge, this association has not been previously reported. Nevertheless, the mechanisms discussed above provide a plausible explanation, as elevated PP may exacerbate BBB disruption and vasogenic edema, while larger infarct volumes associated with higher periprocedural PP may also contribute to cytotoxic edema.

On the other hand, the only hemodynamic factor other than PP that remained independently associated with poor clinical outcomes in our multivariable logistic model was a >40% MAP drop. No such association was observed for PP variability or for pre- or intraprocedural SBP values. Hypotension during MT is a well-recognized risk factor for unfavorable outcome, and a large (>40%) MAP decrease from baseline is one of the most consistent findings across observational studies [5,29,30,31,32]. Previous reports have shown periprocedural PP variability to be independently associated with 90-day functional outcomes [8,33]. In contrast, we did not identify any significant associations between PP variability measures previously reported (CV, MSC, DMM) and any of the outcome measures evaluated in our cohort. In the study by Maïer et al., the association between PP variability and functional outcome disappeared in the GA subgroup, which is consistent with the anesthesia modality in our population [8]. The authors speculated that this may result from medical interventions during induction and maintenance of GA. This aligns with data showing that, under GA, physiologic autonomic BP fluctuations are attenuated, and intraoperative BP variability largely reflects anesthetic management and vasopressor use rather than intrinsic cardiovascular regulation [34]. Although GA does not seem to increase the technical accuracy of BP measurement, it provides a more stable physiological environment with fewer motion artifacts and abrupt autonomic fluctuations, reducing short-term variability unrelated to hemodynamic physiology.

Some studies suggest that PP may be a better predictor of poor outcome in AIS patients than SBP [14,15]. Our results are in line with this observation, as the association between SBP decrease from baseline and outcome disappeared after adjustment for potential confounders, whereas PP remained statistically significant. In the study by Jiang et al., which evaluated BP during the first 24 h after EVT, the predictive power and strength of the association between mean PP and prognosis were superior to those of SBP or DBP, with the highest adjusted odds ratios and best diagnostic performance (AUC = 0.661, 95% CI 0.617–0.705). Moreover, mean PP was closely associated with both unfavorable 90-day outcomes (mRS > 3) and secondary outcomes, including mortality and sICH [14]. Another report showed that PP in the first 72 h after stroke onset was a stronger predictor than SBP, DBP, or MAP for major vascular events and stroke recurrence [15]. Our data provide additional support for the notion that PP may be a more informative prognostic marker of poor AIS outcome than SBP, DBP, or MAP alone.

A notable aspect of our analysis is the prognostic performance of median intraprocedural PP. As a single predictor, median PP demonstrated only limited, albeit statistically significant, discriminative ability for poor 3-month outcome (AUC 0.613), indicating that PP alone is insufficient for reliable risk stratification. However, when added to a multivariable model incorporating well-established clinical and procedural predictors, median PP provided incremental prognostic information: the AUC increased from 0.774 to 0.789, and overall model fit improved significantly in the likelihood ratio test. Although the absolute gain in AUC was modest and did not reach statistical significance, such small changes are common when extending already well-performing models and may still be clinically relevant, particularly for individual risk estimation in high-risk populations. The significant improvement in model fit suggests that PP captures aspects of hemodynamic risk not fully reflected by conventional variables such as age, baseline NIHSS, or reperfusion metrics. These findings support the concept that intraprocedural PP should not be viewed as a standalone prognostic marker, but rather as a complementary, potentially modifiable hemodynamic parameter that can refine risk assessment when integrated into a broader multiparametric model.

The value of PP measurement lies primarily in prognostic stratification: patients with higher intraprocedural PP may warrant closer hemodynamic monitoring, early post-procedural neuroimaging, and more cautious blood pressure management, given their higher risk of unfavorable outcomes and hemorrhagic complications. Thus, PP serves as an adjunctive risk indicator rather than a determinant for patient selection.

Importantly, PP was analyzed as a continuous exposure variable, which avoids arbitrary dichotomization and the loss of statistical power inherent to threshold-based grouping. Thus, the descriptive mRS-based comparisons do not drive the main conclusions; instead, the multivariable regression models and ROC-based analyses directly quantify the independent and incremental contribution of PP to clinical and radiological outcomes. The additional table reporting PP-stratified characteristics further complements these results and addresses the reviewer’s request for PP-oriented descriptive data. After adjustment for multiple comparisons using the Benjamini–Hochberg false-discovery rate correction, the overall pattern of associations remained consistent, supporting the robustness of the findings.

A strength of our study is the procedural homogeneity, as all patients underwent mechanical thrombectomy under GA for proximal anterior circulation LVO (ICA or M1) within a single standardized workflow. However, several important limitations must be acknowledged. First, this was a retrospective, single-center observational study, which precludes any causal inference and carries an inherent risk of selection bias. Second, the potential for residual or unmeasured confounding cannot be excluded, even after multivariable adjustment. Third, blood pressure was measured non-invasively, which is less precise than invasive arterial-line monitoring and may introduce measurement noise or underestimate rapid PP fluctuations. Fourth, although general anesthesia provides a more physiologically stable environment with fewer motion artifacts and abrupt autonomic changes, it does not improve the technical accuracy of BP measurement. Fifth, detailed information on anesthesia management (e.g., anesthetic agents, vasopressor titration) was not available, which may influence intraprocedural hemodynamics. Furthermore, the generalizability of our findings to patients undergoing thrombectomy under conscious sedation, as well as to those with posterior circulation strokes, remains uncertain, as both populations may exhibit distinct hemodynamic responses and autoregulatory profiles. Finally, no formal internal validation (e.g., bootstrapping or cross-validation) was performed to assess coefficient stability or quantify potential model optimism, and therefore the predictive contribution of PP should be interpreted as exploratory. Therefore, the findings of this study should be considered hypothesis-generating and interpreted with caution, and they warrant validation in larger prospective, multicenter studies with standardized hemodynamic monitoring.

## 5. Conclusions

In patients with proximal anterior circulation LVO treated with MT under general anesthesia, higher intraprocedural PP was independently associated with worse 90-day functional outcome, larger FIV, MBE, and HT. Both median PP and the cumulative time spent above PP thresholds (>50, >60, and >80 mmHg) showed a relationship with unfavorable EVT outcome and HT. Among other hemodynamic variables, only a >40% drop in MAP from baseline remained an independent predictor of poor outcome.

These findings suggest that the pulsatile component of blood pressure, beyond absolute SBP or MAP alone, may provide additional prognostic information during EVT. Intraprocedural PP was more strongly associated with FIV, MBE, HT, and 90-day poor functional outcomes than SBP, which warrants further validation of intraprocedural PP as a prognostic biomarker in larger prospective, multicenter studies.

## Figures and Tables

**Table 1:** Baseline characteristics and treatment outcomes.

Total Patients	mRS 0–2 (*n* = 87)	mRS 3–6 (*n* = 130)	*p*-Value	*q*-Value (BH)
Age, years (median, IQR)	68 (60–74)	74 (68–82)	**<0.001 ^1^**	**0.004**
Female, N (%)	53 (61%)	62 (48%)	0.071 ^2^	0.115
Age > 80 years, N (%)	7 (8%)	39 (30%)	**<0.001 ^2^**	**0.004**
**Medical history**				
Hypertension, N (%)	65 (75%)	112 (86%)	0.048 ^2^	0.092
Hyperlipidemia, N (%)	37 (43%)	45 (35%)	0.256 ^2^	0.344
Atrial fibrillation, N (%)	43 (49%)	81 (62%)	0.069 ^2^	0.115
Diabetes mellitus, N (%)	19 (22%)	33 (25%)	0.627 ^2^	0.698
Chronic heart failure, N (%)	10 (11%)	33 (25%)	**0.015 ^2^**	**0.034**
CAD, N (%)	12 (14%)	28 (22%)	0.159 ^2^	0.238
Smoker, N (%)	8 (9%)	10 (8%)	0.803 ^2^	0.824
Active cancer, N (%)	4 (5%)	2 (2%)	0.221 ^2^	0.319
**Baseline characteristics**				
Baseline NIHSS (mean ± SD)	15.4 ± 5.1	18.2 ± 4.7	**<0.001 ^3^**	**0.004**
Onset-to-groin time, minutes (median, IQR)	260 (195–298)	253 (216–300)	0.525 ^1^	0.620
Groin-to-reperfusion time, minutes (median, IQR)	51 (34–80)	73 (48–115)	**<0.001 ^1^**	**0.004**
Bridging thrombolysis, N (%)	64 (74%)	92 (71%)	0.758 ^2^	0.799
Most proximal occlusion, N (%)				
MCA M1	68 (78%)	85 (65%)	0.050 ^2^	0.093
Tandem occlusion	17 (20%)	47 (36%)	**0.010 ^2^**	**0.024**
**Hemodynamic parameters**				
Baseline blood pressure variables, mmHg (median, IQR)				
SBP	150 (133–165)	156 (134–170)	0.242 ^1^	0.337
DBP	83 (79–95)	80 (75–95)	0.604 ^1^	0.693
MAP	104 (97–114)	107 (97–120)	0.693 ^1^	0.751
Median of intraprocedural blood pressure variables, mmHg (median, IQR)				
SBP	115 (110–125)	120 (110–127)	0.370 ^1^	0.465
DBP	70 (65–75)	70 (63–73)	0.055 ^1^	0.098
MAP	87 (81–93)	85 (80–90)	0.304 ^1^	0.395
Pulse pressure variables, mmHg (median, IQR)				
Baseline	60 (50–72)	70 (50–82)	0.086 ^1^	0.134
Maximal	70 (60–80)	80 (62–90)	**0.005 ^1^**	**0.015**
Minimal	40 (30–45)	35 (30–40)	0.919 ^1^	0.919
Median	48 (40–55)	50 (45–60)	**0.008 ^1^**	**0.022**
Coefficient of variation	0.22 (0.15–0.31)	0.21 (0.16–0.28)	0.434 ^1^	0.529
Maximal successive change	20 (18–30)	25 (20–35)	**0.010 ^1^**	**0.024**
Difference maximum–minimum	30 (25–45)	40 (30–53)	**0.027 ^1^**	0.059
Time with pulse pressure over threshold, minutes (median, IQR)				
>50 mmHg	25 (15–45)	45 (20–70)	**<0.001 ^1^**	**0.004**
>60 mmHg	10 (5–23)	20 (5–40)	**0.002 ^1^**	**0.007**
>70 mmHg	5 (0–10)	5 (0–19)	**0.002 ^1^**	**0.007**
>80 mmHg	0 (0–5)	5 (0–5)	**0.004 ^1^**	**0.013**
**Imaging treatment outcome measures**				
Successful reperfusion, N (%)	81 (93%)	108 (83%)	0.038 ^2^	0.078
HT, N (%)	13 (15%)	68 (52%)	**<0.001 ^2^**	**0.004**
PH2	0 (0%)	26 (20%)	**<0.001 ^2^**	**0.004**
sICH, N (%)	0 (0%)	20 (15%)	**<0.001 ^2^**	**0.004**
MBE, N (%)	3 (3%)	49 (38%)	**<0.001 ^2^**	**0.004**

Bold font indicates *q* < 0.05 (Benjamini–Hochberg adjusted *p*-values for multiple comparisons within this Table). mRS, modified Rankin Scale; CAD, coronary artery disease; NIHSS, National Institutes of Health Stroke Scale; ICA, internal carotid artery; MCA, middle cerebral artery; TICI, Thrombolysis in Cerebral Infarction scale; HT, hemorrhagic transformation; sICH, symptomatic intracranial hemorrhage; MBE, malignant brain edema. ^1^ Mann–Whitney U test; ^2^ Fisher’s exact test; ^3^ Student’s *t* test.

**Table 2:** Intraprocedural pulse pressure metrics stratified by key clinical outcomes.

Malignant Brain Edema (MBE)				
Pulse Pressure Metric	Yes	No	*p*-Value	*q*-Value (BH)
Baseline	75 (55–84)	63 (50–80)	0.046	0.103
Maximal	80 (65–90)	70 (60–85)	0.133	0.215
Minimal	38 (30–45)	35 (30–40)	0.330	0.408
Median	50 (45–60)	50 (40–55)	0.046	0.103
Coefficient of variation	0.21 (0.15–0.26)	0.22 (0.16–0.31)	0.079	0.138
Maximal successive change	27 (20–39)	25 (20–35)	0.144	0.216
Difference maximum–minimum	40 (25–50)	35 (25–50)	0.635	0.702
**Any hemorrhagic transformation**				
**Pulse pressure metric**	**Yes**	**No**	***p*** **-value**	***q*** **-value (BH)**
Baseline	70 (60–80)	60 (50–80)	0.052	0.103
Maximal	80 (70–89)	70 (60–85)	**<0.001**	**0.011**
Minimal	40 (30–45)	35 (30–40)	0.027	0.103
Median	50 (45–60)	45 (40–55)	**<0.001**	**0.011**
Coefficient of variation	0.23 (0.15–0.29)	0.21 (0.16–0.28)	0.748	0.785
Maximal successive change	28 (20–39)	23 (20–30)	0.030	0.103
Difference maximum–minimum	40 (30–53)	35 (25–50)	0.054	0.103
**Symptomatic intracranial hemorrhage (sICH)**				
**Pulse pressure metric**	**Yes**	**No**	***p*** **-value**	***q*** **-value (BH)**
Baseline	80 (69–88)	63 (50–80)	**0.005**	**0.035**
Maximal	84 (74–90)	73 (60–86)	0.043	0.103
Minimal	35 (30–41)	35 (30–40)	0.990	0.99
Median	50 (45–60)	50 (40–55)	0.180	0.252
Coefficient of variation	0.22 (0.21–0.29)	0.22 (0.15–0.29)	0.411	0.48
Maximal successive change	30 (25–39)	25 (20–35)	0.247	0.324
Difference maximum–minimum	45 (39–55)	35 (25–50)	0.040	0.103

Bold font indicates *q* < 0.05 (Benjamini–Hochberg adjusted *p*-values for multiple comparisons within this Table. Data are presented as median (IQR). For binary outcomes (mRS 3–6, MBE, any hemorrhagic transformation, and sICH), between-group comparisons were performed using the Mann–Whitney U test. *p*-values were adjusted for multiple comparisons using the Benjamini–Hochberg procedure across all tests reported in this table and are presented as q-values (BH). Abbreviations: mRS, modified Rankin Scale; MBE, malignant brain edema; sICH, symptomatic intracranial hemorrhage; FIV, final infarct volume; PP, pulse pressure; MSC, maximal successive change; DMM, difference maximum–minimum.

**Table 3:** Multivariate logistic regression analyses for poor clinical outcome, malignant brain edema, hemorrhagic transformation, and symptomatic intracranial hemorrhage.

Variable	Poor Outcome (mRS 3–6)			MBE		
	OR	CI 95%	*p*-value	OR	CI 95%	*p*-value
Maximal PP [per 10 mmHg]	1.19	0.99–1.43	0.057	1.08	0.91–1.28	0.403
Median PP [per 10 mmHg]	1.47	1.09–1.99	**0.012**	1.39	1.03–1.86	**0.030**
Maximal successive change [per 10 mmHg]	1.11	0.88–1.41	0.369	1.09	0.88–1.38	0.430
Difference maximum–minimum [per 10 mmHg]	1.11	0.93–1.33	0.245	0.97	0.81–1.16	0.755
PP over threshold [per 5 min]						
>50 mmHg	1.08	1.01–1.15	**0.030**	1.01	1.00–1.13	**0.050**
>60 mmHg	1.10	1.00–1.19	**0.041**	1.04	0.97–1.11	0.296
>70 mmHg	1.03	1.00–1.06	0.086	1.09	0.96–1.23	0.181
>80 mmHg	1.59	1.16–2.19	**0.004**	1.06	0.83–1.37	0.631
	**Any hemorrhagic transformation**			**sICH**		
	OR	CI 95%	*p*-value	OR	CI 95%	*p*-value
Maximal PP [per 10 mmHg]	1.25	1.06–1.47	**0.009**	1.16	0.91–1.48	0.221
Median PP [per 10 mmHg]	1.67	1.25–2.24	**0.001**	1.27	0.82–1.96	0.281
	1.18	0.96–1.45	0.116	1.24	0.92–1.67	0.167
Difference maximum–minimum [per 10 mmHg]	1.22	0.96–1.32	0.156	1.16	0.91–1.48	0.221
PP over threshold [per 5 min]						
>50 mmHg	1.08	1.02–1.14	**0.007**	1.06	0.97–1.16	0.224
>60 mmHg	1.11	1.04–1.19	**0.003**	1.06	0.97–1.16	0.219
>70 mmHg	1.25	1.10–1.42	**<0.001**	1.11	0.94–1.32	0.228
>80 mmHg	1.40	1.10–1.79	**0.006**	0.99	0.68–1.47	0.987

Bold font indicates statistical significance. In the analysis of the association between each parameter and the outcome, the multivariable logistic regression model was adjusted for NIHSS score at admission, age > 80 years, hypertension, chronic heart failure, bridging thrombolysis, onset-to-groin time, groin-to-recanalization time, and the presence of successful recanalization. MBE, malignant brain edema; sICH, symptomatic intracranial hemorrhage; PP, pulse pressure.

**Table 4:** Multivariable linear regression analyses for final infarct volume.

Hemodynamic Variable	B	CI 95%	*p*-Value
Maximal PP [per 10 mmHg]	8.32	(−2.02)–18.68	0.114
Median PP [per 10 mmHg]	18.78	1.55–36.02	**0.033**
Maximal successive change [per 10 mmHg]	5.15	(−8.36)–18.66	0.453
Difference maximum–minimum [per 10 mmHg]	2.38	(−7.97)–12.72	0.650
PP over threshold [per 5 min]			
>50 mmHg	1.13	(−2.15)–4.40	0.499
>60 mmHg	1.22	(−2.83)–5.27	0.555
>70 mmHg	3.55	(−4.23)–11.32	0.369
>80 mmHg	5.59	(−9.63)–20.80	0.470

Bold font indicates statistical significance. In the analysis of the association between each parameter and the outcome, the multivariable linear regression model was adjusted for NIHSS score at admission, age >80 years, hypertension, chronic heart failure, bridging thrombolysis, onset-to-groin time, groin-to-recanalization time, and the presence of successful recanalization. MBE, malignant brain edema; sICH, symptomatic intracranial hemorrhage; PP, pulse pressure.

## Data Availability

The data presented in this study are available on request from the corresponding author. The data are not publicly available due to privacy restrictions.

## Abbreviations

The following abbreviations are used in this manuscript:

AIS	Acute ischemic stroke
AUC	Area under the curve
aOR	Adjusted odds ratio
BBB	Blood–brain barrier
BP	Blood pressure
CBF	Cerebral blood flow
CED	Cerebral edema
CI	Confidence interval
CT	Computed tomography
CV	Coefficient of variation
DBP	Diastolic blood pressure
DMM	Maximum–minimum difference
ESO	European Stroke Organization
EVT	Endovascular treatment
FIV	Final infarct volume
GA	General anesthesia
HT	Hemorrhagic transformation
ICA	Internal carotid artery
IQR	Interquartile range
IVT	Intravenous thrombolysis
LVO	Large vessel occlusion
MAP	Mean arterial pressure
MBE	Malignant brain edema
MCA	Middle cerebral artery
mRS	Modified Rankin Scale
mTICI	Modified Thrombolysis in Cerebral Infarction
MT	Mechanical thrombectomy
NCCT	Non-contrast computed tomography
NIHSS	National Institutes of Health Stroke Scale
PH2	Parenchymal hematoma type 2
PP	Pulse pressure
ROC	Receiver operating characteristic
SBP	Systolic blood pressure
SD	Standard deviation
sICH	Symptomatic intracranial hemorrhage
SITS-MOST	Safe Implementation of Thrombolysis in Stroke–Monitoring Study
Yesss	Trial of Org 10172 in Acute Stroke Treatment
